# Supplementary material for: Exposure to Gulf war illness-related chemicals exacerbates alcohol-induced liver damage in rodents
Source: Sci Rep. 2024 Jul 1;14:14981. doi: 10.1038/s41598-024-65638-5 (PMC11217429; doi:10.1038/s41598-024-65638-5)
Supplement: Supplementary file 1 — Supplementary Information. [file 41598_2024_65638_MOESM1_ESM.docx]

**Supplemental information**

**Exposure to Gulf war illness-related chemicals exacerbates alcohol-induced liver damage in rodents**

**Anca D Petrescu^1,2^, Julie Venter ^1,2^, Daria D Danilenko^1^, Daniela Medina^4^, Stephanie Grant^1,2^, Su Yeon An^1,2^, Elaina Williams^1,2^, Patrick Mireles^1^, Kathryn Rhodes^1^, Matthew Tjahja^3^, Sharon DeMorrow^1,2^**

**Supplemental Table 1**. Statistical analysis of mRNA fold changes for proinflammatory cytokines in livers of Naïve and GWI mice when treated with ethanol vs controls. p- values from student’s ttests when comparing Naïve vs GWI, Naïve+ETOH vs Naïve, GWI+EtOH vs GWI, Naïve+EtOH vs GWI and GWI+EtOH vs Naïve+EtOH, are shown. *, p<0.05, N=4.

| Cytokine | Naïve vs GWI | Naïve+EtOH vs Naive | GWI+EtOH vs GWI | Naïve+EtOH vs GWI | GWI+EtOH vs Naïve+EtOH |
| --- | --- | --- | --- | --- | --- |
| IL-1β | 0.0335 * | 0.0381 * | 0.0272 * | 0.0371* | 0.0371* |
| IL-6 | 0.0123 * | 0.0016 * | 0.0771 | 0.9933 | 0.0555 |
| CCL2 | 0.4295 | 0.1607 | 0.0490 * | 0.2434 | 0.1399 |
| TNFα | 0.0158 * | 0.0467 * | 0.0016 * | 0.0011* | 0.0039* |


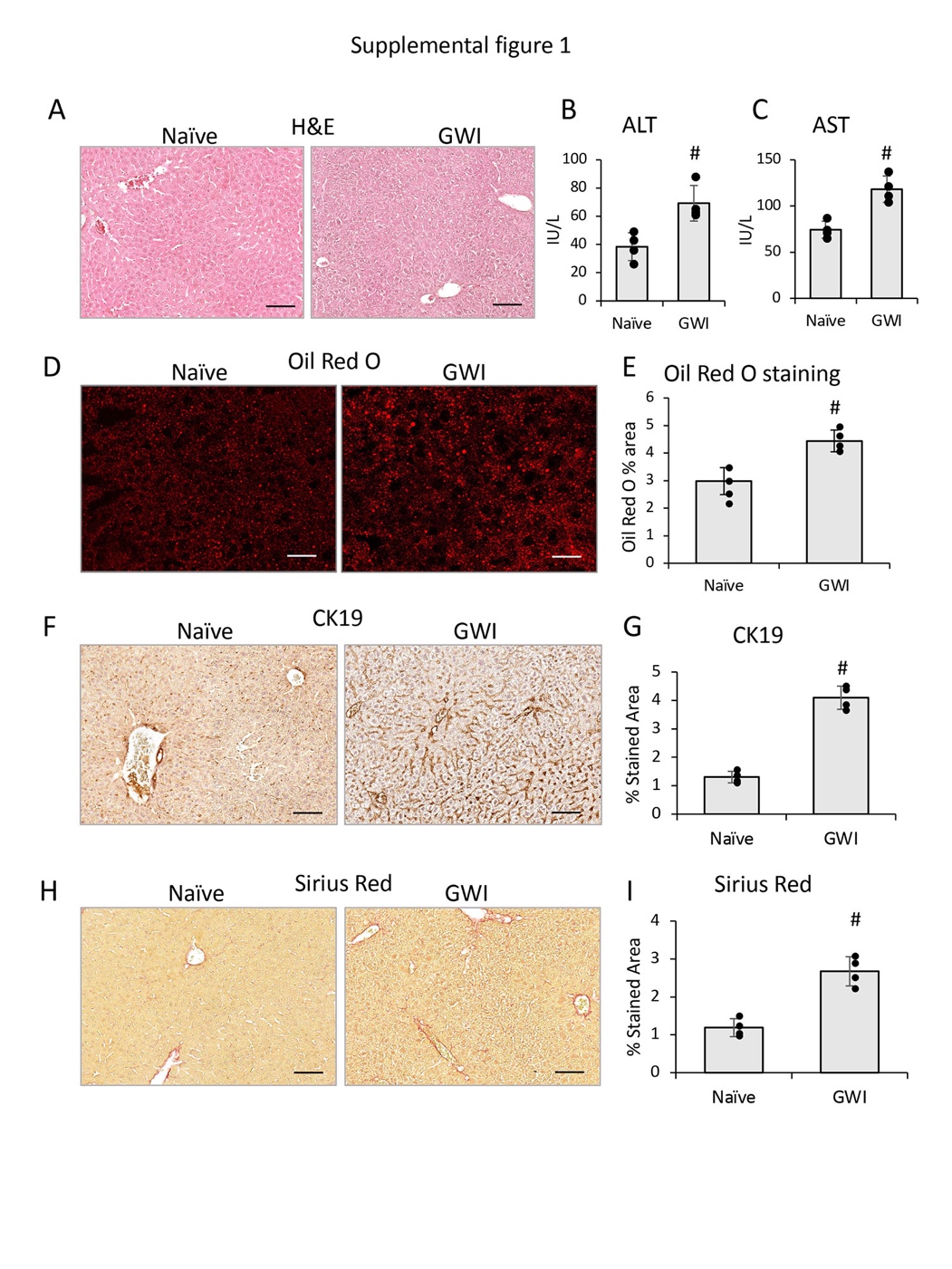


**Supplemental figure 1**. Liver pathology assessment in GWI mice that underwent one year recovery after exposure to GW-chemicals. (A), Images of H&E staining of liver sections from GWI vs Naïve mice. (B), (C), serum level of ALT and AST n GWI vs Naïve mice. (D), (E), Images and quantification of Oil Red O-staining of lipid droplets in livers of GWI vs Naïve mice. (F), (G), Images and quantification of CK19 in cholangiocytes of livers from GWI vs Naïve mice. (H), (I), Images and quantification of Sirius Red-stained fibrillar proteins in livers of GWI vs Naïve mice. p<0.05, N=4. #, GWI vs Naïve mice. Scale bar, 50 μm.


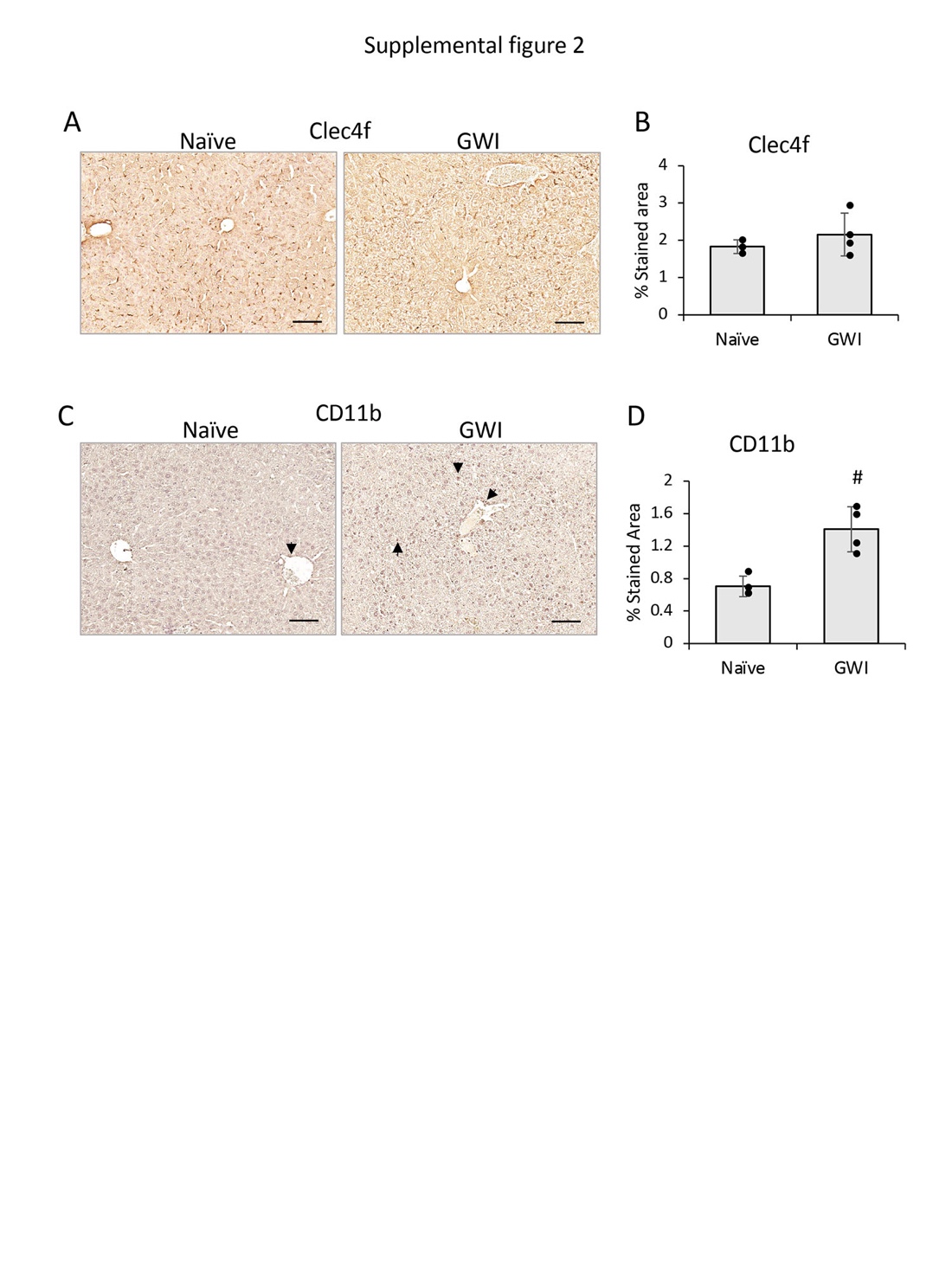
**Supplemental figure 2.** Immunohistochemistry of Kupffer cells and monocyte-derived macrophages in livers of GWI mice that underwent recovery for one year after exposure to GW-chemicals. (A), (B), Images and quantification of Clec4f biomarker of Kupffer cells in GWI mice vs Naïve controls. (C), (D), Images and quantification of CD11b biomarker of macrophages derived from blood recruited monocytes. The arrows point to these very small cells. N=4, p<0.05. #, GWI vs Naïve mice. Scale bar, 100 μm.

**
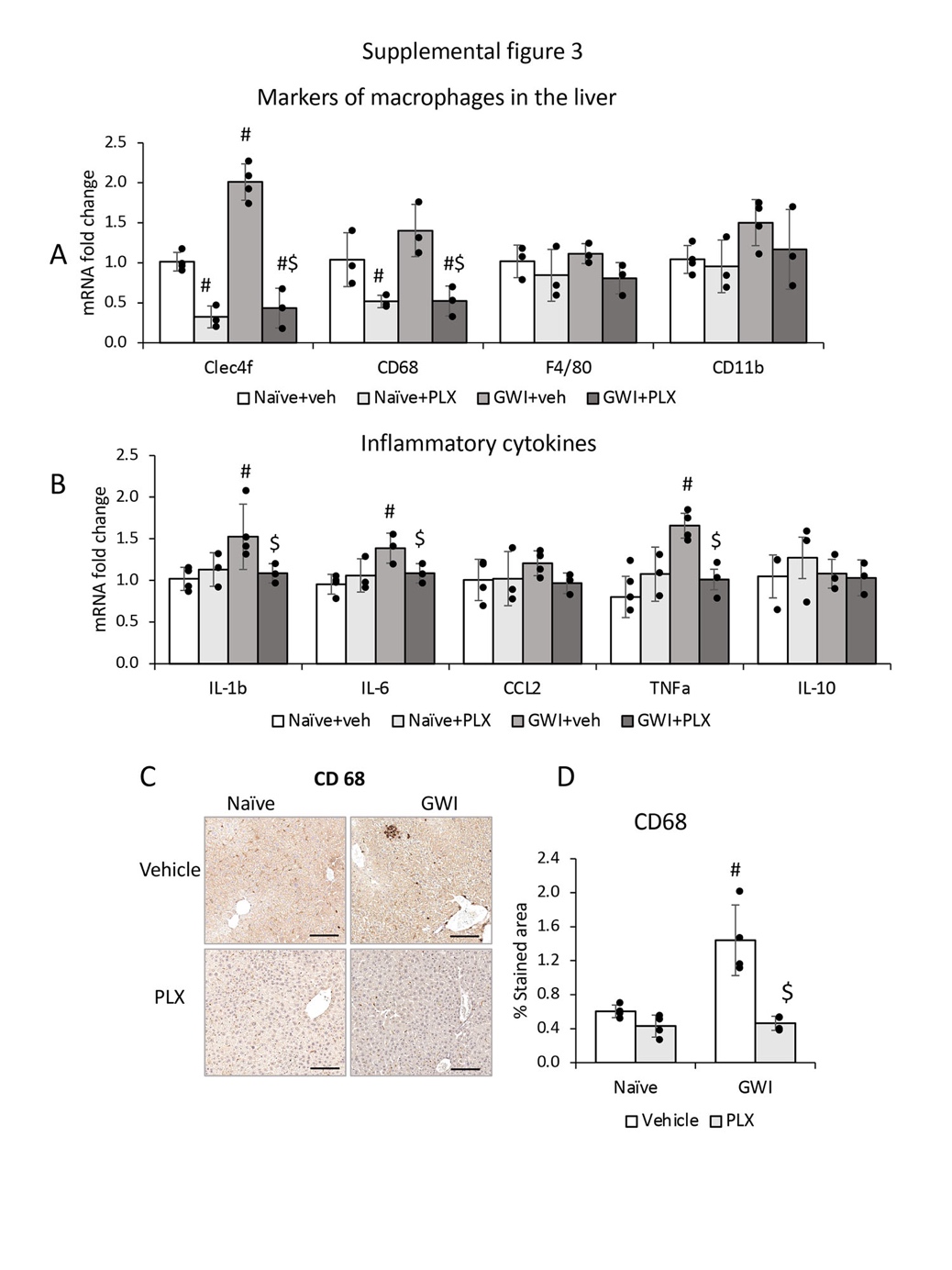
**

**Supplemental figure 3.** The effect of PLX3397 on the expression of genes involved in liver inflammation GWI vs Naïve mice. (A), Changes in mRNA level of biomarkers of macrophages, i.e., Clec4f, CD68, F4/80, CD11b in liver samples from four groups of mice. i.e.: Naïve+vehicle, Naïve+PLX3397, GWI+vehicle, GWI+PLX. (B), Liver mRNA levels of proinflammatory cytokines: IL-1β, IL-6, CCL2, TNFα and IL-10 in same groups as in (A). (C), Images of CD68 IHC in liver sections of Naïve and GWI mice that were treated with vehicle or PLX3397. (D), Quantification of percent area of CD68 staining. N=4, p<0.05. #, vs Naïve with vehicle. $, vs GWI mice. Scale bar, 50 μm.


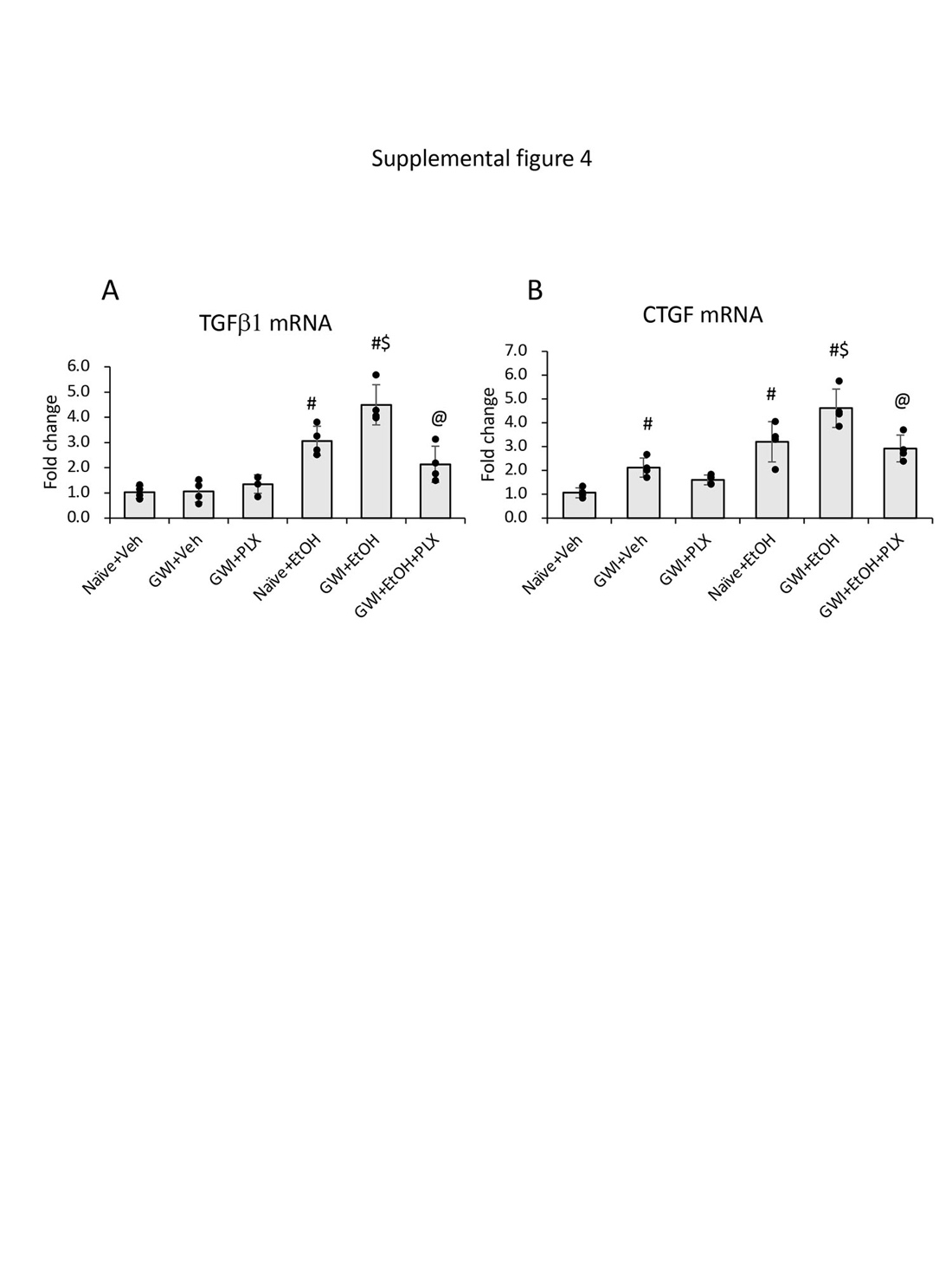


**Supplemental figure** **4**. The effect of EtOH and PLX3397 on expression of fibrogenic genes, in the liver of GWI vs Naïve mice. Changes in TGFβ1 and CTGF mRNA’s were measured using RT-qPCR in livers from the following groups: Naïve mice, with no PER/PB, nor EtOH/PLX; GWI mice were exposed to PER+PB only; GWI+PLX, mice that were exposed to PER+PB, and after recovery received PLX3397, in absence of EtOH; Naïve+EtOH, mice that underwent chronic one time binge of EtOH; GWI+EtOH, mice exposed to PER/PB and then received EtOH; GWI+EtOH+PLX, mice that were exposed to PER/PB and then received PLX3397 followed by EtOH treatment. N=4, p<0.05. #, vs Naïve + vehicle. $, vs GWI. @, vs GWI + EtOH.
